# Brazilian Children’s Understanding of the Quality of Life in Their Living Environment: A Qualitative Study

**DOI:** 10.3390/ijerph17145101

**Published:** 2020-07-15

**Authors:** Camilla Aparecida Silva de Oliveira, Andréa Maria Duarte Vargas, Fernanda de Morais Ferreira, Efigênia Ferreira e Ferreira

**Affiliations:** 1 Department of Social and Preventive Dentistry, Universidade Federal de Minas Gerais, Belo Horizonte 31270-901, Minas Gerais, Brazil; vargasnt@task.com.br; 2 Department of Pediatric Dentistry, Universidade Federal de Minas Gerais, Belo Horizonte 31270-901, Minas Gerais, Brazil; femoraisfe@gmail.com

**Keywords:** Quality of Life, child, qualitative research, urban population, child welfare

## Abstract

(1) Objective: To understand the perception of Brazilian children about the Quality of Life (QoL) considering their living environment. (2) Methods: This is a qualitative study conducted with children aged 6–10 years, from a medium-sized Brazilian municipality, recruited from public and private schools. An adaptation of the “draw, write, and say” method was used to collect data. At first, all children (*n* = 252) drew a “neighborhood with QoL”. On the same day, the researcher analyzed the graphic elements of the representations and intentionally selected the two best-detailed drawings from each class (*n* = 49) and the children were invited to narrate them. The narratives were analyzed through content analysis. (3) Results: Two major themes emerged from the content analysis, namely, the physical environment and social environment. The first included the needs to live in a community, such as housing, places of leisure, essential services, and natural elements. The second was relationships with family and friends. (4) Conclusion: The children presented the meaning of an environment with QoL, pointing out essential items to have this ideal environment. The social environment and the physical environment were perceived interdependently; that is, any change in one of these aspects may affect children’s QoL.

## 1. Introduction

The World Health Organization (WHO) conceptualizes Quality of Life (QoL) as “the individual’s perception of their position in life in the context of the culture and value system in which they live and concerning their goals, expectations, standards, and concerns” [1]. It is a very different concept in the literature but has common aspects. The first is that it must be a multidimensional construct, with the need to include several aspects of life [2]. The second highlights the subjectivity of QoL. Therefore, it can be assessed considering the individual’s perception [3].

Wallander, Schmitt, and Koot [4] reinforce subjectivity, defining QoL as “the combination of objective and subjective wellbeing in multiple domains of life that are relevant in the culture and time of individuals, and which comply with universal human rights’ standards”. Therefore, it is necessary to obtain information directly from individuals [5], that is, we should create a space for the children to speak up [6] in order to analyze children’s QoL.

When discussing subjectivity in manifestations about QoL, Minayo et al. [7] report that they can answer “how people feel or what they think about their lives, or how they perceive the value of the material components recognized as the social basis of QoL”.

Although subjectivity is a strong aspect, many QoL measures designed to measure children’s perception considered the answers provided by relatives or close adults [8,9], which can interfere with the result since the perception of adults about quality life among children does not always accurately reflect the opinions and ideas of this population [4,10]. Some studies indicate that there may be disagreements between the perception of children and their proxies [11,12,13]. Most of the time, the proxy, who is a parent, can report aspects of a child’s behavior, but not the children’s inner thoughts and feelings [5,11].

It is necessary to understand the children’s perspective on their experiences and environment [5] to understand children’s needs. Qualitative research is one way of ensuring that the investigated experiences are being analyzed exclusively from the perspective of the individuals [14]. However, qualitative studies that explore children’s perspective of QoL are scarce, and few geared to this end have been published [15]. Most studies explore QoL from the perspective of the wellbeing of healthy children [16], understanding the impact of different health conditions [17] and specific diseases or physical condition [3,14,18,19]. Not even surveys aimed at the construction of questionnaires to measure children’s QoL identified what a quality life is to children, or the importance attributed to different states of life [4,15,20,21].

It is believed that the scarcity of qualitative studies with this population is associated with the belief that children’s understanding to grasp their world is limited, or they have a hard time expressing their experiences verbally and conceptually. Thus, data obtained in these surveys would be doubtful and invalid [5,10]. However, some reports already affirm that children can be active participants in the research process and can accurately report their experiences [3,14,17]. It is necessary to understand the context in which children are familiar and manage the power difference between children and the researcher—who is an adult—to facilitate the children’s approach, which can be achieved by choosing methods that enable children’s participation and make them feel part of the research process, ensuring the maximum opportunity for them to provide their opinions [5,10].

The other aspect of QoL that must be considered is multidimensionality. We can observe that the QoL construct was strongly influenced by the WHO concept of health, which has a biopsychosocial approach [14]. Considering this approach, QoL must include, at least, the physical, mental, and social domains of health [4,15]. However, if we consider the expanded concept and the social determinants of health, we should direct our lenses to the children’s environment and context since they can influence children’s health and development [22]. However, most QoL instruments already proposed for children do not consider them, and reports of experiences in their environment are also rarely explored [5]. Furthermore, little is known about the health and wellbeing of children growing up in small- and medium-sized urban centers, although it is where most children will grow up and live as adults [23].

Given the above, considering the possibility of building a generic QoL questionnaire for children, we sought to ensure the subjectivity of the QoL construct by conducting a qualitative study with children. We intended to understand the perception of Brazilian children about QoL considering their living environment.

## 2. Materials and Methods

This study was conducted in a medium-sized Brazilian municipality, from September 2017 to June 2018, chosen intentionally for having characteristics similar to most of the Brazilian urban population and for the easy access to children.

We opted to know the children’s perception of a quality neighborhood, through the strategy of drawings with narrative [24], which was a good option since children’s drawings tend to represent what children think about a particular personal, social, or natural reality, sometimes allowing access to conceptual thoughts [25]. Furthermore, according to Vygotsky [26], in contrast to the common sense of insurmountability between them, reality and imaginative activity show connection forms. Most of the time, imagination does not come from nothingness and always results from lived experiences. These real experiences can derive from the imagination, from isolated, recombined elements or emotional expressions, but always lived previously. Imaginative activity is based on reality and previous experience.

The study population consisted of children aged 6–10 years, living in urban areas in the municipality of the study. The entire study was carried out in elementary schools, considering that children’s school attendance is mandatory by law in this age group [27]. This study was conducted in four public schools and one private school in the city to achieve a better representation of Brazilian children. The number of schools and children was defined considering the proportionality of children enrolled in the public (90%) and private networks (10%) in the city [28].

Public schools were selected from their territorial location in the municipality (headquarters and district) and the absolute value of the Basic Education School Socioeconomic Indicator—ESSI (2011/2013), carried out by the Anísio Teixeira National Institute of Educational Studies and Research [29]. In this scenario, two schools from each municipality territory (with higher and lower ESSI) were invited to participate in this research, which allowed children of different socioeconomic conditions to participate in the study, ensuring the variability of the participants concerning this criterion.

All selected schools included classes from the early elementary school years (first to fifth grades), where children aged 6–10 years are found. A class for each elementary school grade was drawn in each school to secure the participation of children of all age groups. All children in these classes were invited to participate and took home the Informed Consent Form (ICF). Only those who returned with the informed consent form duly authorized by the responsible person participated in the data collection. This project was ethically approved by the Research Ethics Committee of the Federal University of Minas Gerais (CAAE: 68497017.0.0000.5149).

Only the leading researcher developed the data collection activities, using a “creative” research method to allow children to express their ideas regardless of their verbal skills and literacy level [24]. Moreover, the method implemented is favorable in developing the child’s trust and a good relationship with the researcher [10]. This method was an adaptation of the “draw, write, and tell” (DWT) method proposed by Angel et al. [24]. It is child-centered, supports thought processes, and allows people to communicate using various resources. Furthermore, it ensures that children expose their interpretation of the drawings, by “saying”, preventing the researcher from providing adult interpretations of the drawing [24]. In this study, we chose not to ask the child to write phrases or name the images, as those under the age of eight could have difficulty with writing since the literacy of the Brazilian population can last up to this age group [30].

All children authorized to participate in the research were directed to a classroom or spaces provided by the school, such as the library. At first, the researcher held a conversation with the children, making it clear that she was not a teacher, and that despite being an adult, she was a student who was researching a good life for children and who was there to learn. Afterward, all the children were invited to make a drawing, following this command: “*Draw a neighborhood that has QoL, a good life, a happy life*”. The material resources provided by the researcher during the activity were a white sheet of legal size A4 paper (21 × 29.7 cm), and a set of hydrographic pens. Children who wished to use rulers, pencils, or their pens were not prevented. There was no time restriction for the development of the activity. However, the children used about 30 to 40 min.

After the end of the activity, the drawings were collected, and the children returned to their classroom. On the same day, the researcher analyzed the graphic elements of the representations and intentionally selected [31] the two best-detailed drawings from each class. It was considered that the children who were more detailed in their drawings would provide better information [31], so they were invited to talk individually and privately about their drawing. It was estimated that the number of approached children would be sufficient to achieve data saturation. In fact, the content found proved to be consistent for the analysis, even before the narratives were finished (saturation by data), allowing the understanding of the patterns, categories and the variety of the studied phenomenon [31].

As an ice-breaker, the researcher made a positive comment about the drawing and asked the child: “*I asked you to draw a neighborhood with QoL, a good life. Tell me what you drew in this neighborhood*”. Then, the researcher guided the child through the drawing so that he could explain each graphic element drawn, without being limited by predefined questions. The narratives of the drawings lasted up to 20 min (average = ten minutes), were audio-recorded (smartphone), and later transcribed in full. The statements were identified by the letter “C” of “child” with the number of the respondent (C1 to C49), followed by the school numbering (S1 to S5) to ensure anonymity. Before the baseline study, the leading researcher undertook theoretical training and a pilot study, under the supervision of qualitative research experts, to adapt the conduct of the narratives.

### 2.1. Classification of Drawings

The drawings of all children who participated in the study were considered for this analysis. At first, the main researcher verified which graphic elements each child had represented in their drawing, categorizing them into physical objects, social objects, and abstract objects [32]. Then, these data were analyzed using frequency distribution and compared between boys and girls using the chi-square test. This stage of the study allowed only to identify which were the most drawn graphic elements by the children [33]. There was no interpretation of the meanings of the drawings by the researchers. This interpretation was performed in the group of children who participated in the narrative of the drawings.

### 2.2. Analysis of the Narratives of the Drawings

A methodological reference for the analysis of the narratives of the drawings was used, which was an adaptation of the content analysis proposed by Graneheim and Lundman [34], offering a systematic way to group data into themes. The unit of analysis was each interview, which was read several times over to understand and apprehend the content and, afterward, the meaning cores were extracted. Subsequently, the meaning cores were condensed, keeping the meaning of the text. Next, each core was interpreted, giving rise to the categories of analysis, which were grouped into the central themes. This process was carried out independently by the leading researcher and the other researchers, qualitative research experts. The categories and themes were established after discussion among all researchers, aiming at a consensus. The analysis was driven by the data and not by the authors’ judgment.

## 3. Results

### 3.1. Characteristics of the Municipality and of Population of Schools

The municipality where the present study was conducted has 84% of urban dwellings with basic sanitation, 66.8% are located on public roads with afforestation and 22.2% on public roads with adequate urbanization (presence of manhole, sidewalk, pavement, and curb) [28].

Students from schools participating in this study differed in relation to socioeconomic status [29]. According to ESSI [29], in schools 1 and 4 (ESSI = 49.33 and 49.54, respectively), a large part of the students were inserted in families having an income of between US $145.92 and US $292.67, parents had low education (incomplete or complete elementary school), and children had access to basic appliances and housing (color television, radio, refrigerator, cell phone, two bedrooms, and a bathroom) and complementary appliances and services (VCRs or DVDs, washing machines, computers, and internet access). In schools 2 and 3 (ESSI = 57.49 and 56.20, respectively), the students’ families had a higher income of between US $292.67 to US $975.58, more elementary, complementary, and also supplementary appliances (freezers, one or more landline phones, and a car). At school E, most of the children (ESSI = 65.44) had a family income of between US $975.58 and US $1365.81, parents who had completed high school, in addition to a higher number of elementary, complementary, and supplementary appliances [29].

### 3.2. Graphic Elements of the Drawings

A total of 252 children were included in the study, with 109 (43.3%) girls and 73 (29.0%) boys attending public schools and 23 (9.1%) girls and 47 (18.6%) boys attending private schools.

**Figure 1:**
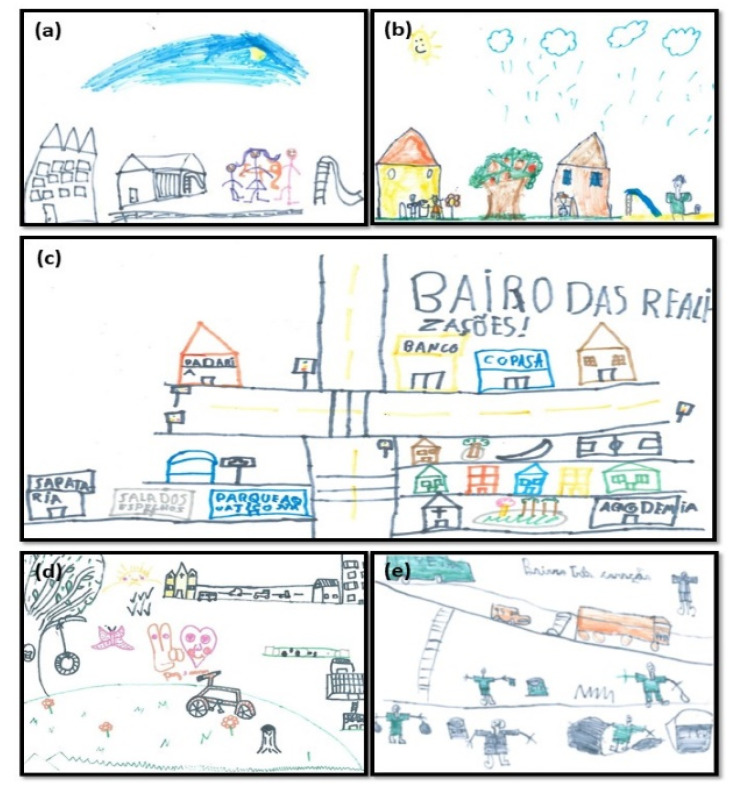
Representative drawings of the Quality of Life (QoL) neighborhood, Brazilian children, aged 6–10 years, urban area. (**a**) C1-S4: 6-year-old boy, (**b**) C4-S3: 7-year-old boy, (**c**) C6-S5: 8-year-old boy, (**d**) C7-S1: 9-year-old boy, (**e**) C8-S3: 9-year-old-boy. C: child; S: school.

**Figure 2:**
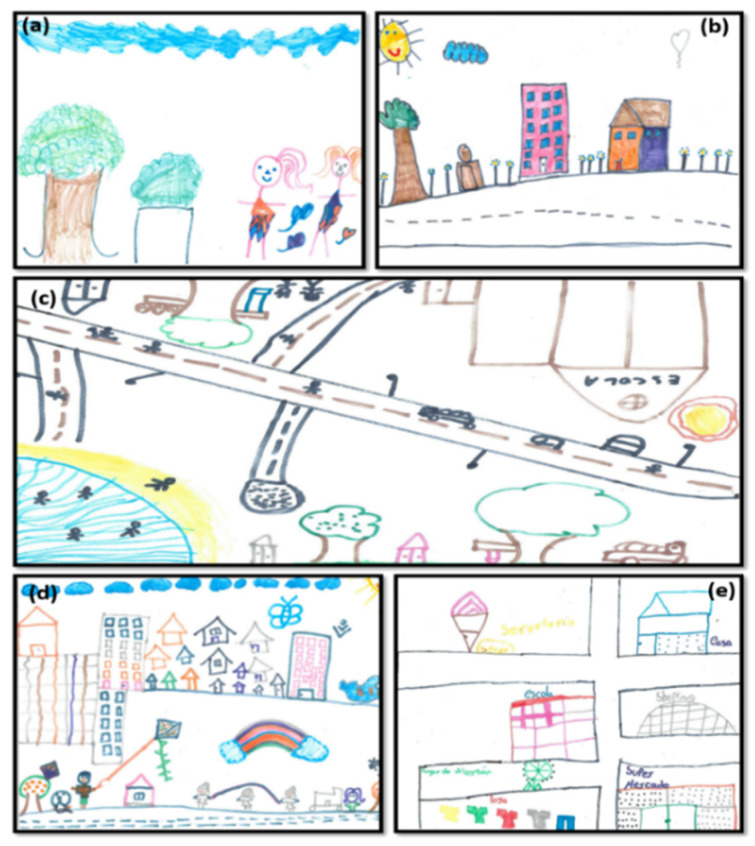
Representative drawings of the Quality of Life (QoL) neighborhood, Brazilian children, aged 6–10 years, urban area. (**a**) C1-S3: 6-year-old girl, (**b**) C4-S5: 7-year-old girl, (**c**) C5-S1: 8-year-old girl, (**d**) C7-S4: 9-year-old girl, (**e**) C7-S1: 10-year-old-girl. C: child; S: school.

When performing the analysis of the graphic elements of the drawings (*n* = 252), it was observed that the children represented physical objects such as natural elements (88.5%), houses (80.2%), buildings (32.5%), means of transport (33.3%), toys (36.1%), places of leisure (28.2%), commerce (17.9%), urban cleanliness (15.1%), school (15.9%), health services (11.1%), and street lighting (8.3%), social objects (57.9%), represented by people, and abstract objects (7.1%), represented, for example, by the symbol of “Peace and Love” (Figure 1 and Figure 2).

The graphic elements represented by both girls and boys showed similarities. The only differences observed between the genders were related to the elements of nature (trees, flowers, lakes, rivers, sun, rain, clouds, birds, and butterflies) and objects related to urban cleanliness (dumps, selective collection, sewage treatment station), drawn more by girls (*p* = <0.05) (Table 1).

Although children were not asked to name the graphic elements or write about their design, it was observed that children eventually used this resource. Some expressed positive feelings in relation to the designed environment, writing phrases such as “It is very nice to live here” (Boy, 8 years old) or named the neighborhood and its streets using expressions such as: “Happy street”, “Victory street”, “Happy neighborhood”, “Fulfillment neighborhood”, “Victory neighborhood”, “Happy as it can be neighborhood”. Awareness messages were also observed, such as “We must not make cities dirty” (Girl 1, 10 years, S4). “We must not litter the city nor the school, please keep it clean” (Girl 2, 10 years, S4).

### 3.3. QoL Themes and Categories

In order to understand the meaning that children gave to graphic elements, the narratives of the drawings were performed. In this stage, a total of 49 children participated, being 24 (49.0%) girls and 15 (30.6%) boys from public schools, and five (10.2%) girls and five (10.2%) boys, from private schools. The proper distribution of the participants concerning age was observed: nine children aged six and ten children in each of the other ages (7 to 10 years). The number of children included in the qualitative sampling was considered sufficient, as data saturation was reached.

From the analysis of the transcriptions, we aimed to understand the meaning of objects for children, and themes and categories emerged, as described in Table 2.

The first topic, the physical environment, pointed out essential attributes of the neighborhood, in the children’s perception, to achieve QoL. This included housing, leisure facilities, essential services, and elements of nature. 

Statements related to housing show the need for a home structure with minimal conditions of comfort, hygiene, and adequate shelter:

*“A clean house that doesn’t have […] a lot of animals […] for example lizards, mice, rats […]” (C3-S2, boy, 7 years old)*

*“A house that is not falling down, like a simple house, but tidy, a house that has no mess […]” (C8-E1, girl, 10 years old)”*

*[…] it has to have a bathroom, living room, kitchen, bedroom […]” (C6-S5, boy, 8 years old).*

Regarding leisure places, the children pointed out that a neighborhood with QoL must have squares, parks, courts, soccer fields, and clubs. In their view, these environments are structured places to have fun and play. In addition, they promote social interaction with friends and family and contact with elements of nature, such as plants, trees, and lakes. The following statements demonstrate these thoughts:

*“I made a square for the children to have fun […] there are plants, toys and a place for them to play.” (C7-S5, girl, 9 years old)*

*“A town also has to have a leisure area […] to have a picnic, for children to play and have fun.” (C6-S5, boy, 8 years old).*

*“The park is good for spending time with the family, they will have some fun”. (C10, S5, boy, 9 years old).*

*“A square […] For parents and children to interact more, have a better life, run, play on the swing, slide, ride a bike.” (C9, S5, girl, 10 years old).*

*“The court is for children to have a place of leisure […] as it is close to the neighborhood, they could go with their friends to play.” (C9, S2, boy, 9 years old).*

*“The square is for people to sit down […] there is a lake […] there is a tree, there are benches […]” (C5, S5, girl, 8 years old).*

The children pointed out several urban attributes that are important for the QoL in the neighborhood. Among these, the essential services that are guaranteed by law, such as collective public transport, street lighting, urban cleanliness, food stores, banking services, health facilities, and security are highlighted. It is noteworthy that, although more girls have drawn graphic elements that represent urban cleanliness, no differences were identified in their speeches when compared to boys.

*“It’s a bus. It doesn’t matter, we have a house and we have to walk a lot to catch the bus, then it doesn’t do it, especially when we are late” (C8-S3, boy, 9 years old).*

*“A light pole. If there is no light, everything is dark at night, then you cannot see.” (C5-S1, girl, 8 years old).*

*“I drew […] the garbage dumps […] if it [the neighborhood] is not clean […] we are getting sick […] it can bring several bacteria and dengue mosquitoes.” (C8-S3, boy, 9 years old).*

*“The trash is not to let the neighborhood very dirty.” (C5-S2, girl, 8 years old).*

*“The supermarket…because we buy […] things to eat.” (C8-S1, girl, 10 years old).*

*“We have to use the bank often, […] all of these (things) are there for us not to go far, for us to have access.” (C6-S5, boy, 8 years old).*

*“A hospital […] when someone gets sick or has a problem. You just take them to the hospital, and they are taken care of.” (C7-S5, girl, 9 years old).*

*“If someone was sick […] if we didn’t have a health post, he could die.” (C5-S2, girl, 8 years old).*

Ensuring public safety in the neighborhoods is essential to achieve the children’s desire to live in a quiet and violence-free neighborhood:

*“Nobody fights with anyone; there’s no shooting, there are no thugs. Everyone is calm, in their corner.” (C7-S1, boy, 9 years old).*

*“Love […] the real neighborhood has to have this […] you have to sit and talk, […] make things up.” (C9-S4, girl, 10 years).*

*“A neighborhood that has nothing wrong […] that doesn’t have much movement on the streets” (C8-S4, boy, 9 years old).*

Although this study did not seek to understand the reality that children live, it was observed in their speeches that public security can be ineffective, since feelings of danger and insecurity in relation to their living environment were pointed out. It is assumed that this perception is being included by family members, since children expose in their reports the parents’ view of the danger in their environment:

*“Because if I go alone [to the park], […] some men […]. some bikers would catch me […] I will only go when my mother goes.” (C1-S4, boy, 6 years old).*

*“I don’t leave the house because my mother doesn’t let me […] she said that I can play inside or outside on the balcony […] on the street not […] because there may be cars and buses.” (C2-S4, girl, 6 years old).*

*“No, I can’t [fly a kite on the street] because my mother is afraid of the bad people in my neighborhood […] the bad guys” (C8-S4, boy, 9 years old).*

These negative experiences may have influenced the children’s perception, which made them look for resources that would increase the security of their homes, the neighborhood and the streets. The children drew watchmen, policemen, guard dogs, high walls, bars on the windows, and reported that the presence of these elements could inhibit violent acts in the neighborhoods:

*“[…] Many people can’t jump over the wall, because the wall is high […] there was a dog there […] they barked too much and helped us because a thug wanted to steal some things from the little room […]”. (C3-S2, boy, 8 years old).*

*“Because if someone gets in, the dog will bite.” (C8-S4, boy, 9 years old).*

*“[…] Then like a dog, it just likes the owner, then if another person is going to do bad things, then it goes there and bites them […] It can help me.” (C9-S4, girl, 10 years).*

*“[…] There are three watchmen […] at night they have a motorcycle, each one goes to a neighborhood […] so as not for people to steal, so that bad things don’t happen”. (C5-S5, girl, 8 years old).*

*“Having more security, having more policemen in the neighborhood going around […]” (C7-S1, boy, 9 years old).*

The children also pointed out that some resources in the neighborhood could increase their safety in traffic:

*“[…] without traffic signs […] a car could run over […] These are pedestrian crossings […], and accidents don’t happen.” (C6-S5, boy, 8 years old).*

Although the school is an environment frequented by all the children in this study, it was observed that this space was poorly designed and explored. Only two points were found in the children’s speeches: the school must be close to home and it is a place of learning that will collaborate with the child’s future.

*“The school is the place that teaches children. So, it is good for making children smarter and …and improve in the future, to raise awareness.” (C10-S5, boy, 10 years old).*

*“So children don’t have to leave their neighborhood to study […].” (C9-S2 boy, 10 years old).*

For children, a neighborhood with QoL must have elements of nature, such as trees, plants, flowers, rivers, lakes, sun, and rain. In this study, it was more common for girls to draw these elements. However, there were no differences in children’s perception based on gender. Both are aware of the importance of the environment for maintaining terrestrial life and the need to preserve it:

*“Rain is important for plants to grow.” (C3-S3, girl, 7 years old).*

*“Rain is good for plants.” (C4-S3, boy, 7 years old).*

*“[…] It’s water, and they also go to rivers and sewers, it becomes clean for us to drink and wash the pots, the bathroom.” (C8-S2, girl, 9 years old).*

*“The house needs water […] without it, nothing works […]” (C2-E1, boy, 6 years old).*

*“For when you are cold, the sun warms you up. If you are homeless.” (C7-S4, girl, 9 years old).*

*“The sun […] provides light and warmth” (C7-S1, boy, 9 years old).*

*“The sun has a vitamin […] staying. in the sun before 10 a.m. is very good, but then […]” (C9-S4, girl, 10 years old).*

*“The tree makes us breathe properly […] The air is good for us to breathe […]” (C4-S5, girl, 8 years old).*

*“For us to have some shade and breathe.” (C6-E4, boy, 8 years old).*

*“[…] Some trees bear fruits, and fruits are healthy for us […]” (C2-S5, girl, 6 years old).*

*“[…] They bear fruits that are good for health, for example, orange, apple […]” (C3-S2, boy, 7 years old).*

*“Because if you don’t take care of them [trees], they can die.” (C1-E3, girl, 6 years old).*

*“oh, to preserve nature … also because there are a lot of people cutting trees.” (C7-S2, boy, 9 years old).*

*“I designed few cars because the smoke pollutes the environment […] I did it there more to demonstrate liveliness, which is in need” (C7-S1, boy, 9 years old).*

*“[…] Cars [produce] a lot of smoke and pollute the air.” (C8-E2, girl, 9 years old).*

The second theme, the social environment, pointed out the importance of social interaction with family and friends to obtain a quality life. In addition to the family being associated with positive feelings, affection, and care, the children were able to understand that parents are fundamental to guarantee their basic needs:

*“We have to love our family a lot […] they help us do everything […] study […] work to buy our little things.” (C1-S3).*

*“[…] Taking care of us with affection, love, kisses, and hugs.” (C4-S2).*

Relations with friends were also frequently commented on. In speeches, friends were always present in games and conversations. Furthermore, it was with them that children shared their things.

*“[…] it’s good to have friends […] we lend and share things.” (C1-S1).*

*“Me and my friend […] we can talk to them about many things we don’t like, that we are ashamed to tell our dad and mom.” (C9-S4).*

*“I like to send a lot of emoji to my friends.” (C10, S3, girl, 10 years old).*

*“Play hide and seek, high pike, colorful elephant” (C3-S5).*

*“[…] I like to go to her house, because […] she plays with me.” (C3, S3, girl, 7 years old).*

### 3.4. Theoretical Model

**Figure 3:**
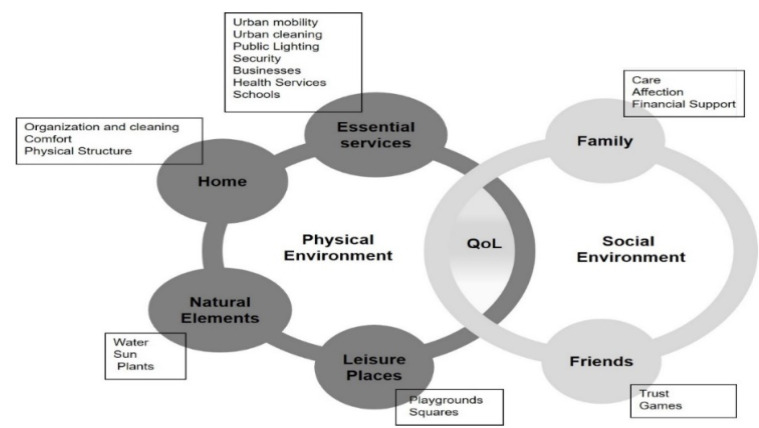
Conceptual Quality of Life (QoL) model for children in their living environment.

Figure 3 illustrates a conceptual model of QoL, in the child’s living environment, built from the concepts generated in the narratives of the drawings. All the attributes pointed out by the children are important to obtain QoL. The social environment and the physical environment are interdependent. Changes in any of the attributes of the physical environment can ignite the social environment, or vice versa. For example, it was possible to observe in the children’s quotes that the lack of security in the neighborhood triggers feelings of fear and danger within the family. This fact makes children use the leisure facilities in the neighborhood less and consequently may have reduced social interaction with friends.

## 4. Discussion

This study aimed to understand the meaning of QoL in the environment from the perspective of Brazilian children. It was not in the interest of this study to learn about the reality of children, but how they conceive a good place to live.

Two themes were identified from the narratives of the drawings: the first revealed issues related to the physical environment, showing how the organization of cities and society are essential to achieve QoL, in the children’s perception. The second pointed out the importance of social interaction with family and friends to achieve a quality life.

The first theme reported in this study included the physical environment of the neighborhoods. Besides their own home, children spend most of their time in the neighborhood. Therefore, it is the experiences lived in these places that make them assess which aspects are essential for their lives, and what can be improved in the environment [35,36,37]. In this work, the statements pointed out that housing, essential services, leisure spaces, and natural elements must be available and accessible to the population, and these results also observed in national [38] and international [37] studies.

Statements related to housing show the need for a home structure with minimal conditions of comfort, hygiene, and adequate shelter. These characteristics are fundamental in supporting the wellbeing of residents and are perceived by children as aspects that interfere with QoL [39].

Children mentioned different urban services, categorized as “essential services” since they are fundamental to human life. Possibly, it was the positive or negative experiences of users that made them realize how important these places are [37], or more used by families [40]. Public transport, street lighting, and cleaning, services such as school, health facilities, and shops were some items reported.

It must be remembered that although they may not be aware of the text of the Brazilian Constitution, these children’s manifestations are described in Chapter II, *On social rights*, Article 6, and are internationally recognized as universal human rights:

*Article 6. Education, health, food, work, housing, transportation, leisure, security, social security, maternity and child protection, assistance to the destitute are social rights, in the form of this Constitution* [41].

One cannot talk about QoL without assuring these rights, and children already understood this. However, none of the items in this category were actively explored by the children, not even the school, the primary environment in the children’s context [42]. Other studies have observed this fact [16,37], showing the need to explore the school context better and better understand its influence on QoL.

The combination of structural elements with nature also stood out in this study. Most of the children drew elements such as trees, plants, flowers, rivers, lakes, sun, and rain. As observed in the literature [37,43], their statements showed a strong feeling of protection and appreciation of the natural environment, mainly because they perceive how these resources are fundamental in the preservation of human, animal, and plant life. Some children’s reports denote a perception that may be due to the discussions promoted in the school core since environmental education is a mandatory and permanent component at all levels and modalities of Brazilian schools [44].

Another point to be highlighted is that some children concentrated on the natural elements in an area of their drawing, representing the importance of the presence of green areas in the neighborhoods. Studies point out [45,46] that the presence of large areas with more vegetation is well used by children, which is perceived by them as an improvement factor in children’s QoL.

In the opinion of the children in this study, green areas, together with other public spaces, provide children with leisure time with the development of recreational activities, together with friends and family. According to them, recreational activities and games are essential to obtain better QoL and are favored when the neighborhoods have fields, parks, and playgrounds, preferably well-structured and with playground equipment. These reports corroborate the study [47] and reinforce the idea that characteristics of the neighborhoods influence the development of outdoor games [36], which constitute moments that facilitate the construction of social relationships and the promotion of the community [48].

However, there is no point in having well-equipped public spaces if other factors prevent the child population from using them. Some children report the prohibition of attending some public spaces in the neighborhood or even walking without the presence of an adult [40]. This limitation is usually associated with the parents’ perception of danger in their environment. The parents’ fear, while justified, is transferred to their children, and barriers are involuntarily erected and prevent the establishment of social and trusting relationships with the community [36,49].

The danger in the neighborhood was reported by the children from two perspectives: community violence and safety in traffic. As per the literature [37,50,51], the elaborated statements show that the children show awareness and feelings of concern regarding the hostility experienced in the neighborhoods. Given these experiences, it is not surprising that they seek alternatives that ensure security and curb violent attitudes [40]. The security features pointed out by the children were watchmen (or police on the streets), watchdogs, bars on the windows of their homes, high walls in their homes, and traffic lights. Undoubtedly, the lack of security has an impact on QoL, and, therefore, it is necessary to design neighborhoods that provide safer routes and characteristics for children [36,40,52], assuring the opportunity to live in a society [50].

The emergence of the social environment theme, which encompasses relationships with relatives and friends, is widely explored in studies that measure children’s QoL [53]. Their constant presence can be justified by the fact that relationships are vital to human existence and are part of children’s daily lives [54].

Among these relationships, family relationships appeared as an essential aspect of QoL, which was also observed in the literature [15,16,54]. Children in this study argue that relationships in the family environment must be established by a combination of values, which includes care, affection, and respect. The family is highly relevant to children towards achieving the perceived QoL, and they express this thought. The establishment of a healthy family environment is strongly associated with the feeling of happiness [55], which motivated the addition of the expressions “a good life”, “a happy life” in the proposal made to the children so that they draw a neighborhood with QoL. Exposure to family conflicts, violence, and battery makes children unhappy [15] and adversely affects their daily development and, consequently, leads to poor QoL [55,56].

The children in this research also recognized social relationships outside the family as essential, and frequently drew and commented on the existence of friends. They believe the bond with friends, generated through games, conversations, and attitudes of cooperation and kindness, are essential factors for achieving a quality life. According to Wee, Chua, and Li [15], having friends is a crucial factor in QoL to children, and play is an essential component of social relationships.

The theoretical model obtained in this study reinforced the concept that already exists in the literature [37], which is that the physical and social environments are interdependent. The characteristics of the physical environment can shape the behavior of individuals [57] or vice versa. The children in this study well described some interrelationships. For example, the unsafe physical environment can influence the behavior of children’s families, making them more protective. Given the danger, the tendency is for families to forbid their children to use green and leisure spaces that are accessible in the neighborhood on their own, which ends up disfavoring another aspect of QoL, social relationships. The interaction with friends, through conversations and games, ends up being impaired, that is, the present model also elucidated the idea that the availability and accessibility of resources in urban spaces are essential and simultaneous points to achieve QoL [43].

All categories shown here must be evaluated when planning neighborhoods and cities. If this happens, the child’s perception will be considered, allowing creating well-planned environments that allow them to benefit from their physical and community environment; that is, they will provide friendlier urban environments [43]. The contents identified here reinforced the multidimensional character of QoL [4] and the idea that this construct should be assessed broadly, considering all aspects of someone’s life [58].

Two points of this study deserve to be highlighted. Firstly, despite the intentional inclusion of children from different socioeconomic conditions (ESSI), no selection for any specific condition was performed. All children authorized by their parents and who wanted to participate were included in the study. This characteristic allowed verifying the perception of QoL in the environment in a generic way. This approach was different from other studies [3,14,17,18,19], which focused on assessing the perception of specific diseases or conditions. If the focus of this study were specific conditions, probably other dimensions of the QoL construct would have emerged since other experiences would be being considered [15].

The second point relates to the research methods used. We chose to use a child-centered, participatory research method [47], recognizing the capacity and the right of this population to have a voice in the issues that involve them [14,40]. The use of narratives in the drawings was appropriate for all age groups, as all participants expressed their ideas and verbalized them in an articulate manner and in much detail. As in other studies [47,49], the resource used stirred children’s thinking and facilitated an extensive exploration of the topic, ensuring data credibility [47]. Moreover, we decided to direct the drawings with a broad question, which allowed the children to present their responses and to stimulate spontaneous reactions and provide valuable information on how children understand the issue [37].

### Limitations

Although this study is unprecedented, it is exploratory, and with limitations. The details of all phases of the study (design, data collection, and data analysis) have been presented in this manuscript, however, qualitative research is inherently interpretive, which limits another researcher to replicate and to produce identical findings [59]. This research was developed only with children from the urban space of a single Brazilian municipality, which must be considered when using its results. For comparative purposes, researchers should consider the characteristics of their scenarios and samples, ensuring data transferability to their own context [59]. In this study, it was observed that there are similarities in children’s perception, although they lived in different neighborhoods. However, the expansion of this study to other Brazilian regions could identify different perceptions since the cultural and local contexts may differ. It is up to the researchers, therefore, to further study the theme, discussing the value of the physical environment to children, and the need to include these new categories in the generic measures that assess QoL [53].

## 5. Conclusions

The children drew and presented the meaning of an environment with QoL, pointing out items that they considered essential to have this ideal environment. The social and physical environments were perceived by the children interdependently; that is, any change in one of these aspects may have an impact on children’s QoL. All of the items mentioned refer to basic premises that should exist in a society committed to human rights. It is necessary to consider the multidimensional concept of QoL, and mainly, the necessary subjectivity in studies and evaluations, even in children.

## Figures and Tables

**Table 1:** Comparison of the graphic elements of the drawings between boys and girls, aged 6–10 years old, Brazil (*n* = 252).

Graphic Elements		Girls	Boys	Total	*p*
		*n* (%)	*n* (%)		
**Physical Objects**					
natural elements	Present	125 (56.1)	98 (43.9)	223	**0.001**
	Absent	7 (24.1)	22 (75.9)	29	
houses	Present	107 (53.0)	95 (47.0)	202	0.707
	Absent	25 (50.0)	25 (50.0)	50	
buildings	Present	45 (54.9)	37 (45.1)	82	0.581
	Absent	87 (51.2)	83 (48.8)	170	
means of transport	Present	37 (44.0)	47 (56.0)	84	0.061
	Absent	95 (56.5)	73 (43.5)	168	
toys	Present	45 (49,5)	46 (50.5)	161	0.484
	Absent	87 (54.0)	74 (46.0)	91	
places of leisure	Present	35 (49.3)	36 (50.7)	71	0.539
	Absent	97 (53.6)	84 (46.4)	181	
Commerce	Present	24 (53.3)	21 (46.7)	45	0.888
	Absent	108 (52.2)	99 (47.8)	207	
Urban cleanliness	Present	26 (68.4)	12 (31.6)	38	**0.032**
	Absent	106 (49.5)	108 (50.5)	214	
School	Present	25 (62.5)	15 (37.5)	40	0.162
	Absent	107 (50.5)	105 (49.5)	212	
Health services	Present	14 (50.0)	14 (50.0)	28	0.789
	Absent	118 (52.7)	106 (47.3)	224	
Street lighting	Present	12 (57.1)	9 (42.9)	21	0.648
	Absent	120 (51.9)	111(48.1)	231	
**Social Objects**					
People	Present	77 (52.7)	69 (47.3)	146	0.894
	Absent	55 (51.9)	51 (48.1)	106	
**Abstract Objects**					
Symbols of hearts, and “Peace and Love”	Present	11 (61.1)	7 (38.9)	18	0.442
	Absent	121 (51.7)	113 (48.3)	234	

Note. Significant results at 5% level in bold type.

**Table 2:** Description of the themes and categories observed in the drawings with narrative, about Quality of Life (QoL) in their living neighborhood, 6–10 years old, Brazil.

Themes	Analysis Categories	Description
**Physical environment**	Home	Organized and clean domestic environment, comfortable, physical structure
	Leisure Place	Squares, parks
	Essential services	Urban mobility, urban cleaning, public lighting, shops and health services, schools, security
	Natural Elements	Water, sun, plants
**Social environment**	Family	Care, affection, and financial support
	Friends	Confidence; toys and games
